# Alginate-Lysozyme Nanofibers Hydrogels with Improved Rheological Behavior, Printability and Biological Properties for 3D Bioprinting Applications

**DOI:** 10.3390/nano12132190

**Published:** 2022-06-26

**Authors:** Maria C. Teixeira, Nicole S. Lameirinhas, João P. F. Carvalho, Bruno F. A. Valente, Jorge Luís, Liliana Pires, Helena Oliveira, Martinho Oliveira, Armando J. D. Silvestre, Carla Vilela, Carmen S. R. Freire

**Affiliations:** 1 CICECO—Aveiro Institute of Materials, Department of Chemistry, University of Aveiro, 3810-193 Aveiro, Portugal; maria.teixeira@ua.pt (M.C.T.); nicoleslameirinhas@ua.pt (N.S.L.); joao.pedro.carvalho@ua.pt (J.P.F.C.); bfav@ua.pt (B.F.A.V.); jorge.luis@ua.pt (J.L.); liliana.pires@ua.pt (L.P.); martinho@ua.pt (M.O.); armsil@ua.pt (A.J.D.S.); cvilela@ua.pt (C.V.); 2 EMaRT Group—Emerging: Materials, Research, Technology, School of Design, Management and Production Technologies Northern Aveiro, University of Aveiro, 3720-509 Oliveira de Azeméis, Portugal; 3 Department of Biology & CESAM, University of Aveiro, 3810-193 Aveiro, Portugal; holiveira@ua.pt

**Keywords:** alginate, lysozyme nanofibers, hydrogels, bioinks, rheological properties, extrusion 3D bioprinting, cell-laden scaffolds

## Abstract

In this study, alginate nanocomposite hydrogel bioinks reinforced with lysozyme nanofibers (LNFs) were developed. Alginate-LNF (A-LNF) suspensions with different LNF contents (1, 5 and 10 wt.%) were prepared and pre-crosslinked with 0.5% (*w*/*v*) CaCl_2_ to formulate A-LNF inks. These inks exhibit proper shear-thinning behavior and good recovery properties (~90%), with the pre-crosslinking step playing a crucial role. A-LNF fully crosslinked hydrogels (with 2% (*w*/*v*) CaCl_2_) that mimic 3D printing scaffolds were prepared, and it was observed that the addition of LNFs improved several properties of the hydrogels, such as the morphology, swelling and degradation profiles, and mechanical properties. All formulations are also noncytotoxic towards HaCaT cells. The printing parameters and 3D scaffold model were then optimized, with A-LNF inks showing improved printability. Selected A-LNF inks (A-LNF0 and A-LNF5) were loaded with HaCaT cells (cell density 2 × 10^6^ cells mL^−1^), and the cell viability within the bioprinted scaffolds was evaluated for 1, 3 and 7 days, with scaffolds printed with the A-LNF5 bioink showing the highest values for 7 days (87.99 ± 1.28%). Hence, A-LNF bioinks exhibited improved rheological performance, printability and biological properties representing a good strategy to overcome the main limitations of alginate-based bioinks.

## 1. Introduction

In the last decade, three-dimensional (3D) bioprinting has emerged as a novel biofabrication methodology, with the capacity to generate 3D functional living structures by depositing layer-by-layer cell-laden biomaterials, providing a base for the development of tissue constructs, organs and organoids, and organs-on-chips that mimic natural counterparts [1]. Due to the automated deposition process, 3D bioprinting offers significantly improved control over the architecture of the fabricated tissue constructs with high reproducibility [2]. This technology has gathered monumental attention and has been extensively investigated for wide-ranging applications, such as regenerative medicine, tissue engineering and transplantation, the screening of drugs and cancer research, among others [3,4,5].

This method of 3D bioprinting utilizes bioinks, composed of biomaterials, cells, bioactive molecules (e.g., growth factors) and their combinations [6], to fabricate the cell-laden tissue constructs. The design and optimization of bioinks aims to explore and manipulate artificial biological and biochemical environments that could accommodate and allow the growth of living cells, in combination with suitable rheological and mechanical properties [2]. Hence, the basic aspects of an ideal bioink are mainly associated with biocompatibility and biodegradability, high mechanical integrity and stability, and the capability to foster cell adhesion and proliferation [6]. Among the different types of bioinks, the ones based on hydrogels are the most described and investigated materials [7,8], because their peculiar architecture provides permeability to oxygen, nutrients, and other water-soluble compounds, along with allowing cellular migration and communication within the porous flexible network [9]. Moreover, the selection of the bioprinting technique, for instance, between extrusion-based [10,11], material jetting [12,13], or Vat-polymerization methodologies [14,15], strongly depends on the type of bioink [16], with extrusion bioprinting being the most explored for hydrogels [17]. Hydrogels can be produced with both natural (e.g., cellulose, chitin, alginate, pectin, starch, collagen and fibrin), and synthetic (e.g., polyacrylamide, poly(vinyl alcohol) and poly(2-hydroxyethyl methacrylate)) derived polymers [18,19]. The use of biopolymeric hydrogels, based on polysaccharides or proteins, for 3D bioprinting applications offers several advantages over the synthetic ones [20,21]. Specifically, in addition to their biocompatibility towards mammalian cells and tissues, most biopolymers are biodegraded under physiological conditions, leading to the formation of nontoxic degradation products. Furthermore, biopolymers are considered eco-friendly polymers as a result of their renewable and biodegradable nature [22].

Amid the vast array of polysaccharides, alginate is a polyanionic water-soluble linear polysaccharide extracted from brown algae [23] that forms hydrogels under mild conditions, and almost instantaneously, by ionotropic gelation with divalent cations, such as Ca^2+^ [24]. Actually, alginate hydrogels are one of the most studied biomaterials in the domain of 3D bioprinting [21,25,26], because of their excellent tunability (in terms of viscosity modulation, for instance, by varying the concentration of the biopolymer) and printability (in terms of shear-thinning properties) [24]. Albeit their widely recognized and explored advantages, alginate hydrogels are also known to have some limitations. Particularly, alginate is a relatively inert natural polymer deprived of cell-binding receptors, which does not favor cell adhesion and proliferation [27]. Another limitation of alginate hydrogels is related to some degree of uncertainty in their degradation rates, which depend on the diffusion of the Ca^2+^ in aqueous media and consequent disruption of the gel network [28]. This drawback can affect the long-term mechanical stability of 3D bioprinted constructs [29]. Simple strategies can be used to overcome these limitations, namely by combining alginate with bioactive materials, such as Arg-Gly-Asp (RGD) and Tyr-Ile-Gly-Ser-Arg (YIGSR) cell-adhesive peptides [30] and/or other biopolymers, such as gelatin [31] or carrageenan [32]. Moreover, the production of nanocomposite alginate hydrogels using nanostructured materials has also been explored for the same purposes [33]. Commonly used nanomaterials include inorganic (e.g., magnetic, gold, silver [34]) nanoparticles and carbon nanotubes [35]. Nevertheless, the lack of biocompatibility of some of these materials has raised interest in using biobased nanofibrillar structures [36] particularly cellulose nanostructures, such as cellulose nanofibrils [37], cellulose nanocrystals [38] and bacterial nanocellulose [39], to reinforce alginate-based hydrogel bioinks.

Protein nanofibers, also called amyloid fibers, are self-assembled protein nanostructures, organized in cross-β sheets [40]. Due to their exceptional properties, namely high tensile strength [41] and thermochemical stability [42], protein nanofibers have recently gained increasing interest in the development of innovative functional nanomaterials for different applications, including biosensors, scaffolds, drug carriers and nanocomposite materials [43]. In particular, lysozyme nanofibers (LNFs) obtained from an inexpensive globular protein, present a unique combination of size, aspect ratio, chemical composition, and mechanical strength, which can be translated into materials with significantly improved mechanical performance [44]. Additionally, due to their biological nature, they can add bioactive properties to these materials, such as antioxidant and antimicrobial activities [45], and/or promote cell adhesion and proliferation [44]. However, the use of these nanofibrillar materials to produce bioinks with improved properties is still almost unexplored. To the best of our knowledge, only one study has reported the mechanical reinforcement of alginate hydrogels with amyloid curly fibers [46].

Following this premise, the present work reports the development and characterization of novel nanocomposite alginate-LNF-based hydrogels, to produce bioinks with improved mechanical performance and cell viability. Alginate-LNF inks were characterized regarding their rheological behavior, and the corresponding hydrogels regarding their mechanical performance, degradability, and cytotoxicity towards the human keratinocyte (HaCaT) cell line. Afterwards, printing parameters (nozzle diameter and printing pressure and speed) were optimized, as well as the model of the produced scaffolds (dimensions and line spacing), whose morphology was also characterized. The most promising formulations were loaded with living cells and bioprinted, and the cell viability was evaluated for 1, 3 and 7 days. 

## 2. Materials and Methods

### 2.1. Chemicals and Materials

Acetic acid (≥99.7%), cholinium chloride (≥98%), glycine (≥98.5%), dimethyl sulfoxide (DMSO, ≥99.9%), hen egg white lysozyme (HEWL, ~70,000 U mg^−1^), hydrochloric acid (37% *v*/*v* in solution), phosphate buffer saline (PBS, pH 7.4), sodium alginate (low viscosity, 4–10 cP) and 3-(4,5-dimethylthiazol-2-yl)-2,5-diphenyltetrazolium bromide (MTT, 98%) were purchased from Sigma-Aldrich (Sintra, Portugal). Anhydrous calcium chloride (≥99.9%) was acquired from Carlo Erba Reagents (Val de Reuil, France). Dulbecco’s Modified Eagle Medium (DMEM), fetal bovine serum (FBS) and trypsin/EDTA (0.25%/0.02%) were obtained from PAN-Biotech (Aidenbach, Germany). Penicillin/streptomycin solution (100×) and L-glutamine solution (200 mM) were obtained from Grisp (Porto, Portugal), and fungizone from Gibco^®^ (Life Technologies, Carlsbad, CA, USA). A LIVE/DEAD cell viability kit containing ethidium homodimer-1 (EthD-1) and Calcein AM was purchased from Invitrogen (Carlsbad, CA, USA). Ultrapure water (type 1, 18.2 MΩ·cm resistivity (at 25 °C and 0.5 L min^−1^)) was obtained with a Simplicity^®^ Water Purification System (Merck, Darmstadt, Germany).

The nontumorigenic immortalized human keratinocyte cell line (HaCaT), was obtained from Cell Lines Services (Eppelheim, Germany).

### 2.2. Production of Lysozyme Nanofibers (LNFs)

LNFs with an average width between 33–35 nm were produced following a method developed and described by our research group for the fibrillation of lysozyme (HEWL) using deep eutectic solvents (DES) [42]. Succinctly, HEWL (4 mg mL^−1^) was dissolved in an aqueous buffer solution (10 mM HCl at pH 2 with 20 mM glycine) containing 5% (*v*/*v*) of a DES composed of cholinium chloride and acetic acid ([Ch]Cl:Ac (1:1)), and incubated at 70 °C overnight under magnetic stirring. The obtained LNFs were recovered and washed two times with ultrapure water by centrifugation at 15,000 rpm for 45 min (Thermo Scientific Megafuge 16R centrifuge), and afterwards lyophilized in a Telstar (LyoQuest, Tokyo, Japan) freeze drier for 24 h.

### 2.3. Preparation of Alginate-LNFs Suspensions, Inks and Hydrogels

A 4% (*w*/*v*) alginate solution was prepared in ultrapure water at 60 °C under magnetic stirring for 1 h, for the complete dissolution of the alginate. The alginate-LNF precursor suspensions were prepared by the addition of 1, 5 and 10 wt.% (with respect to the alginate mass) of LNFs to the alginate solution and stirred until complete homogenization. Following this, alginate-LNF inks were prepared by pre-crosslinking the previous suspensions, with the addition of a 0.5% (*w*/*v*) CaCl_2_ solution in a volume ratio of 4:1 (alginate-LNFs:CaCl_2_) and homogenized with the help of a mechanical mixer. The inks were placed in a falcon tube and let to rest overnight. 

Fully crosslinked alginate-LNF hydrogel samples were produced by transferring a portion of each alginate-LNF ink into a cylindrical mold and placing them in a CaCl_2_ solution at 2% (*w*/*v*) for 15 min. Before testing, the dimensions of the samples were measured with a digital caliper, reaching an average diameter of 10 mm and height of 5 mm. Table 1 lists all the samples (suspensions, inks, and fully crosslinked hydrogels) that were prepared in this study.

### 2.4. Cell Culture

Immortalized human keratinocytes (HaCaT cell line) were grown in complete DMEM supplemented with 10% non-inactivated FBS, 2 mM L-glutamine, 10,000 U mL^−1^ penicillin/streptomycin and 250 µg mL^−1^ fungizone, at 37 °C, in a 5% CO_2_ humidified atmosphere. Cells were daily observed under an inverted-phase-contrast Eclipse TS100 microscope (Nikon, Tokyo, Japan), and were used after reaching up to 80% confluence. 

### 2.5. Rheological Characterization of the Alginate-LNF Suspensions and Inks

All rheology experiments were carried out with a Kinexus Lab+ Rheometer (Malvern Instruments Limited, Malvern, United Kingdom) equipped with a Peltier element for temperature control, and a cone-plate geometry with a cone angle of 4° and a diameter of 40 mm, using a water lock to prevent dehydration of the samples.

#### 2.5.1. Shear Viscosity Measurements

The shear viscosity of the alginate-LNF suspensions and inks was evaluated as a function of shear rate at 20 °C by steady shear measurements, with shear rates ranging from 0.01–1000 s^−1^. Experimental viscosity data were adjusted to the power-law model [47] according to the following equation:

(1) $$\eta =K\;{\left(\gamma \right)}^{n-1},$$

where η is the sample shear viscosity (Pa·s), *K* is the consistency coefficient (Pa·s), γ is the measured shear rate in s^−1^, and *n* is the power-law index.

#### 2.5.2. Oscillatory Measurements

The alginate-LNF inks’ yield stress was determined by an oscillatory shear stress sweep test (in the linear viscoelastic range (LVR)) performed at 1 Hz with a shear stress range from 1 to 100 Pa. The yield stress was defined as the shear stress at the crossover point of the storage (G′) and loss (G″) moduli. For the determination of the recovery rate of the alginate-LNF inks, a 3-step oscillatory test was carried out consisting of: (i) measurement of G′ in a relaxation stage at 1 Pa for 1 min; (ii) measurement of G′ in a stress phase at 100 Pa for 10 s; and (iii) a second measurement G′ in a relaxation stage at 1 Pa for 1 min. The initial G′ was defined as the average G′ in the initial 1 min when the material was submitted to 1 Pa of shear stress. The recovered G′ was defined as the average G′ after the shear stress reduced from 100 Pa back to 1 Pa. The percent recovery was defined as the recovered G′ divided by the initial G′ and multiplied by 100, according to the equation:

(2) $$Recovery\;\left(\%\right)=\frac{{G^{\prime }}_{Recovered}}{{G^{\prime }}_{Initial}}\times 100,$$

Both tests were also performed at 20 °C.

### 2.6. Characterization of the Alginate-LNF Hydrogels

#### 2.6.1. Morphology of the 3D-Printed Scaffolds 

The morphological analysis of the 3D-printed scaffolds was performed by scanning electron microscopy (SEM). Prior to the analysis, the printed scaffold samples were freeze-dried for 48 h and then coated with a carbon layer using an EMITECH K950 coating system. The micrographs of the surface of the samples were acquired using a HITACHI SU 70 high-voltage microscope (Hitachi High-Technologies Corporation, Tokyo, Japan) operated at 4.0 kV. The SEM images were processed using the ImageJ software to determine the pore dimensions of the materials. At least 20 measurements were considered for each sample.

#### 2.6.2. Swelling Behavior

The swelling ratio of the fully crosslinked alginate-LNF freeze-dried hydrogels was determined by a general gravimetric method based on the amount of absorbed water. Hydrogel samples were freeze-dried for 48 h, weighed, and then immersed in ultrapure water for 24 h at 37 °C. At different time intervals, namely 0.25, 0.5, 1, 2, 4, 6 and 24 h, each sample was retrieved and re-weighed after gently removing the excess surface water with filter paper. Swelling ratios were calculated using the following equation:

(3) $$Swelling\;ratio\;\left(\%\right)=\frac{W\left(swell\right)-W\left(dry\right)}{W\left(dry\right)}\times 100,$$

where *W*(*swell*) and *W*(*dry*) corresponds to the hydrogel weights in the swollen and dry states, respectively.

#### 2.6.3. Degradation Tests 

Degradation profiles of the alginate-LNF hydrogels in PBS and DMEM were determined. Hydrogel samples were first immersed in ultrapure water overnight to reach the swollen equilibrium state. Afterwards, the hydrogels were weighed and placed in PBS or DMEM for 24 h at 37 °C and, at the selected time points of 0.5, 1, 4, 6, 8, and 24 h, retrieved from both media and weighed again, after excess media removal. The degradation percentage was calculated using the following equation:

(4) $$Degradation\;\left(\%\right)=\frac{W\left(i\right)-W\left(t\right)}{W\left(i\right)}\times 100,$$

where *W*(*i*) is the initial weight of each sample and *W*(*t*) is the weight of the sample at each time point.

#### 2.6.4. Oscillatory Measurements (G′ and G″)

Rheological characterization of the alginate-LNF hydrogels was carried out by performing oscillatory shear strain measurements within the linear viscoelastic region, performed at a frequency of 1 Hz, in a shear strain range from 0 to 100%, and with a measurement gap of 3.5 mm, at 20 °C. Again, hydrogel samples were immersed in ultrapure water overnight to ensure the swelling equilibrium. 

#### 2.6.5. Compression Tests

The mechanical properties of the alginate-LNF hydrogels were evaluated by compressive tests using an INSTRON 5966 Series machine (Instron Corporation, Norwood, MA, USA) equipped with a static load cell of 500 N at a rate of 5 mm min^−1^. All tests were performed at up to 80% of strain. At least 5 cylindrical samples, also in the swollen equilibrium state, were tested for each hydrogel. The YounG′s modulus, compressive stress and compressive strain values at the compressive yield point were calculated using the Bluehill 3 (Version 3.22, Illinois Tool Works Inc., Glenview, IL, USA) material testing software.

#### 2.6.6. In Vitro Cytotoxicity

The cytotoxicity of the alginate-LNF hydrogels extracts towards human HaCaT cells was evaluated following the MTT assay [39], for 24, 48 and 72 h. Two independent assays, with 6 replicates each, were carried out. To prepare the hydrogel extracts (25 mg mL^−1^), previously sterilized samples with two ultraviolet (UV) radiation cycles of 20 min were placed in complete DMEM medium, and then incubated at 37 °C, with 5% CO_2_, for 24 h. 

HaCaT cells were seeded in a 96-well plate, at 6000 cells/well, 4000 cells/well and 2000 cells/well, and exposed to the extracts for 24, 48 and 72 h, respectively. HaCaT cells exposed only to complete DMEM medium were used as the control. At the end of the incubation time, 50 μL of MTT (1 g L^−1^) were added to each well and incubated for 4 h, at 37 °C, in 5% CO_2_ humidified atmosphere. After that, culture medium with MTT was removed and replaced by 150 μL of DMSO, and stirred in an orbital shaker for 2 h, in the dark, to completely dissolve the formazan crystals. The absorbance of the samples was measured with a BioTek Synergy HT plate reader (Synergy HT Multi-Mode, BioTeK, Winooski, VT, USA) at 570 nm, with blank corrections. The cell viability was calculated with respect to the control cells for each time point:

(5) $$Cell\;viability\;\left(\%\right)=\left[\frac{\left(Abs_{sample}-Abs_{DMSO}\right)}{\left(Abs_{control}-Abs_{DMSO}\right)}\right]\times 100,$$

where *Abs_sample_* is the absorbance of the sample, *Abs_DMSO_* is the absorbance of the DMSO and *Abs_control_* is the absorbance of the control.

### 2.7. Printability Tests and Optimization

Alginate-LNF scaffolds were printed using a 3D-Bioplotter printer (Developer Series-EnvisionTEC GMBH, Gladbeck, Germany). The printing tests started with the optimization of the basic printing parameters, such as nozzle diameter, printing pressure and speed. Using different nozzle diameters (0.20, 0.25 and 0.41 mm) with a fixed nozzle height of 0.5 mm, multiple experimental trials were carried out to print single strands with approximately 750 mm length by adjusting two essential printing parameters: printing pressure (ranging 0.5 to 3.5 bar in 0.5 bar increments) and printing speed (ranging 5 to 35 mm s^−1^ in 5 mm s^−1^ increments) using the A-LNF0 ink as model sample. Printed lines were digitally photographed, and their width was measured at three random locations using Fiji image processing software (ImageJ, GNU General Public License).

Subsequently, selected processing parameters (printing pressure of 2 bar and printing speed of 10 mm s^−1^) were tested for printing 3D constructs with all ink formulations using a 0.25 mm nozzle. The 3D scaffold model was designed with the BioCAD software as square grids of 20 mm × 20 mm with a 0–90° deposition direction, and a single layer height of 0.320 mm. Printing resolution was also optimized based on the printed scaffolds of the A-LNF0 ink by varying the crosshatch infill line spacing from 1.5 to 3 mm, and layer numbers between 2 to 10. All samples were printed at 20 °C.

The scaffolds were imaged using a stereomicroscope (Nikon SMZ18, Tokyo, Japan) and images were captured with a camera (SRH Plan Apo 2, Tokyo, Japan). The magnification power of the ocular lens was 5× and that of the objective lens was 1.5×, corresponding to a total magnification of 7.5×. Image acquisition was performed using NIS Elements Imaging Software (4.50.00 (Built 1117), 2017). The scaffolds were imaged immediately after printing, before and after the final crosslinking step. The images were also processed using the ImageJ software for the determination of the pore area (*A*) and perimeter (*L*) of each pore in the scaffold for the different inks. 

The printability of the inks (*Pr*) was determined using the following equation:

(6) $$Pr=\frac{L^{2}}{16A},$$

where *L* is the perimeter of the pores of the printed grid structure and *A* is the area of the pore of the printed grid structure. 

### 2.8. Preparation and Characterization of Cell Laden Alginate-LNF Bioinks and Bioprinting

#### 2.8.1. Bioinks Preparation and Bioprinting

The A-LNF0 and A-LNF5 ink formulations were selected for the bioprinting process. The ink formulations were prepared as described in Section 2.3, under sterile conditions. Cultured cell suspensions were transferred to 50 mL centrifuge tubes and centrifuged for 30 min at 5000 rpm. The cell pellet was resuspended in 1 mL of DMEM, and then added and homogeneously mixed within the inks, before the pre-crosslinking step. The final cell density of the bioinks reached 2 × 10^6^ cells mL^−1^. Before the 3D-bioprinting process, formulations were pre-crosslinked as previously described, and the 3D bioprinter and all accessories were sterilized. Two-layer scaffolds of 20 mm × 20 mm with a line spacing of 2.25 mm were bioprinted using the 0.25 and 0.41 nozzles, with a printing pressure of 2 bar and a printing speed of 10 mm s^−1^. The bioprinted structures were crosslinked with a sterile CaCl_2_ 2% (*w*/*v*) solution for 30 min, then replaced with DMEM and incubated for 7 days. 

#### 2.8.2. Cell Viability

At 1, 3 and 7 days post-bioprinting, the culture media was removed, and the scaffolds were washed two times with sterile PBS, before being stained with the LIVE/DEAD reagents: EthD for labelling dead cells (red) and Calcein AM for labelling live cells (green). The fluorescent dyes were prepared according to the standard protocol provided by the supplier, added to the scaffolds ensuring full coverage and then removed after a 30 min incubation period at 37 °C. Afterwards, sample imaging was performed using a confocal microscope (Zeiss LCM 880, Carl Zeiss, Oberkochen, Germany). The percentage of viable cells was measured also using the image analysis tool, ImageJ. In brief, for each image the EthD and Calcein channels were separated and the red (dead) and green (live) intensities were normalized using a grayscale filter and measured for a pre-determined and standardized designated area. The percentage of viability was calculated as:

(7) $$Cell\;viability\;\left(\%\right)=\frac{green\;intensity}{red+green\;intensity}\times 100,$$

### 2.9. Statistical Analysis

All statistical analyses were performed by considering at least three independent tests (unless stated otherwise). Mean values and standard deviations of obtained data were calculated. Statistical significance was determined using the analysis of variance (ANOVA) and Tukey’s test (OriginPro, version 9.9, OriginLab Corporation, Northampton, MA, USA) with the statistical significance established at *p* < 0.05.

## 3. Results and Discussion

**Figure 1:**
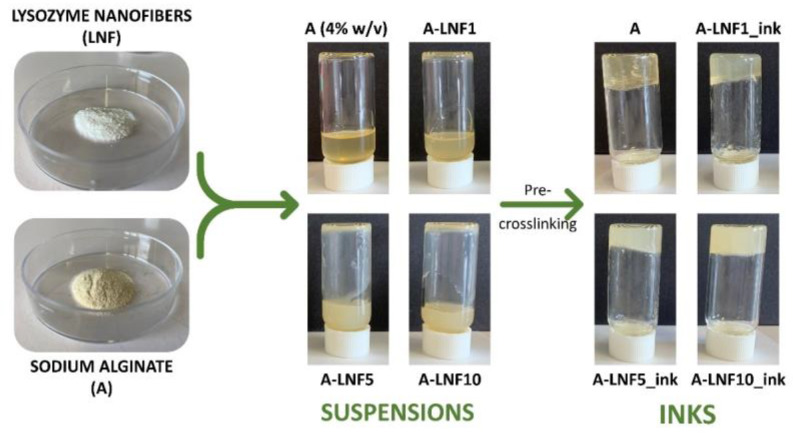
Schematic illustration of the preparation of the ink formulations containing alginate and different amounts of LNFs.

This work focused on the preparation and characterization of novel biopolymeric nanocomposite hydrogel-based bioinks composed of sodium alginate and LNFs for improved 3D bioprinting applications. Several formulations containing alginate (4% (*w*/*v*)) and different amounts of LNFs (1, 5 and 10 wt.%, in respect to the mass of alginate) were prepared and pre-crosslinked with a solution of CaCl_2_ aiming to produce inks with distinct compositions and adequate printability for extrusion 3D bioprinting (Figure 1). All the inks were characterized in terms of their rheological properties, and the corresponding fully crosslinked hydrogels were evaluated regarding their rheology, swelling behavior, degradation, mechanical properties, and cytotoxicity. The 3D-printing process of the developed inks was then optimized to select the best printing parameters (nozzle diameter, printing pressure and printing speed) and print 3D constructs. Finally, selected formulations (A-LNF0 and A-LNF5) were laden with HaCaT cells and 3D bioprinted, and the cell viability after printing was evaluated for 1, 3 and 7 days to assess the ability of the bioinks to be bioprinted without significantly affecting the cell viability, and whether the 3D-printed constructs constitute an adequate environment for cell proliferation.

### 3.1. Rheological Characterization of the Alginate-LNF Suspensions and Inks

**Figure 2:**
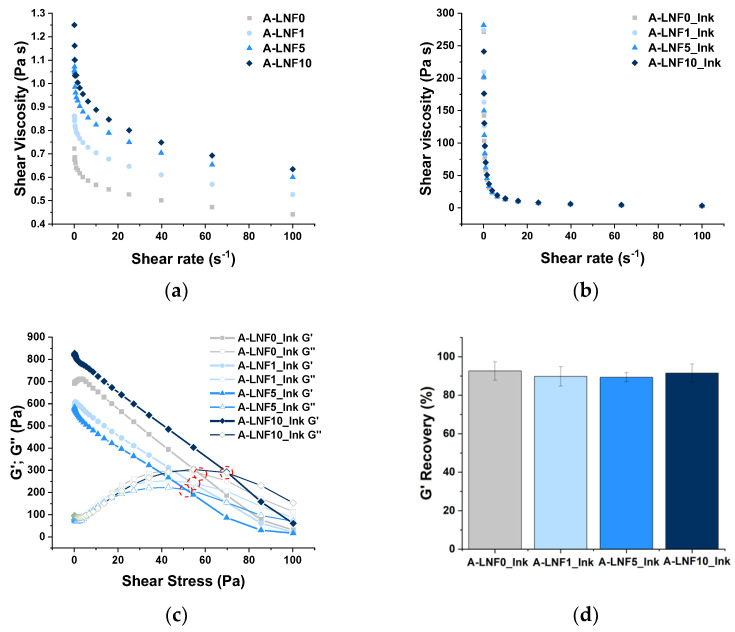
Graphical representation of the (**a**) shear viscosity as a function of shear rate of alginate-LNF suspensions with different contents of LNFs; (**b**) shear viscosity as a function of shear rate of alginate-LNF inks with different contents of LNFs; (**c**) yield stress as a function of shear stress of alginate-LNF inks, and (**d**) G′ recovery percentage of alginate-LNF inks.

The rheological characterization of the alginate-LNF suspensions and inks (pre-crosslinked samples) was carried out, allowing the preliminary assessment of both the printability of the prepared biomaterials and shape fidelity of the printed constructs. During printing, ink formulations should ideally exhibit shear-thinning behavior, i.e., the decrease in shear viscosity with the increase in the shear rate applied, a typical behavior of non-Newtonian fluids [47]. In fact, both alginate solution and alginate-LNF suspensions presented shear-thinning behavior, as observed in the correspondent flow viscosity curves (Figure 2a). It was also observed that the shear viscosity increased with the amount of LNFs. For example, the initial shear viscosity, measured at a shear rate of 0.1 s^−1^ (Table 2), of the pristine alginate solution (A-LNF0, 0.722 ± 0.203 Pa·s) increased 73% with the addition of 10% of LNFs (A-LNF10, 1.250 ± 0.338 Pa·s). To further investigate this issue, the experimental flow viscosity values were adjusted to the power-law model [47], typically used to describe non-Newtonian fluids, based on the calculus of the power-law index (*n*) and consistency coefficient (*K*). Non-Newtonian fluids are characterized by *n* values close to 0 and elevated *K* values [48]. The *n* values of these suspensions approached 1 and the *K* values ranged very low (Table 2). Together with the calculated R^2^, which shows a weak model correlation, these results indicate that the alginate-LNF suspensions exhibited poor shear-thinning behavior.

Consequently, a pre-crosslinking step of the alginate-LNF suspensions, with a small volume of a CaCl_2_ solution at 0.5% (*w*/*v*), was investigated as a strategy to achieve formulations with adequate rheological properties for 3D-extrusion bioprinting, an approach previously exploited for the modulation of alginate-based bioinks [49,50]. The addition of a small volume of this CaCl_2_ solution increased the initial shear viscosity of the alginate solution and of the alginate-LNF suspensions about 300-fold. Despite quite similar shear viscosity values for all formulations (Figure 2b), a slight increase in this parameter (about 24%) is still perceived with the increase in the content of LNFs, specifically from 271.50 ± 29.85 Pa·s (A-LNF0_Ink) to 336.10 ± 84.55 Pa·s (A-LNF10_Ink). These values are within the range of shear viscosity (30 to 522 Pa·s) reported by Habib et al. [51] for inks composed of alginate 4% (*w*/*v*) and CMC 4% (*w*/*v*), where CMC was used as a rheology modifier. Regarding the power-law fitting, the pre-crosslinked samples showed *n* values close to 0 and *K* values raised as the LNF content increased, indicating a more pronounced shear-thinning behavior [48,52]. Moreover, the calculated R^2^ values are also closer to 1 (Table 2), confirming a strong correlation of the experimental data to the power-law model. Therefore, these results indicated that, despite the influence of the LNF content, the pre-crosslinking step was crucial to produce suitable ink formulations based on alginate and LNFs, enhancing their viscosity and shear-thinning performance. This type of strategy has been previously described to enhance the viscosity of alginate-based bioinks reinforced with silk fibroin, to ensure adequate properties for extrusion [50].

The yield stress is another important rheometric parameter that relates to the printability of the inks as it is considered as the minimum stress or force required to initiate the extrusion of the ink through the nozzle [48]. Figure 2c plots the retrieved storage (G′) and loss (G″) moduli as a function of the applied shear stress (Pa) for the inks’ formulations developed in this study. The yield stress was determined as the crossover of the G′ and G″ curves (i.e., when G′ = G″), [53,54], and the obtained values are summarized in Table 2. Yield stress values varied between 50.32 ± 0.49 Pa for A-LNF0_Ink, and 65.63 ± 2.84 Pa for A-LNF10_Ink. These values are lower than those reported by Paxton et al. [48] for inks of 8% alginate (*w*/*v*) pre-crosslinked with CaCl_2_ 1% (*w*/*v*) (166 Pa). This difference is certainly associated with the fact that, in our study, the concentrations of alginate and of the CaCl_2_ solution are half of those used in this previous study.

The recovery ability of a bioink is another characteristic that influences the shape fidelity of the 3D-printed constructs [55] since it relates with the capacity of the ink to recover its initial state, avoiding its spreading after being extruded from the nozzle. To explore this property, a recovery test was performed by monitoring the G′ in three different shear stress stages [53]. The recovery percentage for the alginate-LNF inks was calculated, considering the average values of the initial G′ and the recovered one (Figure 2d). All samples showed similar results, with G′ recovery percentages around 90% (Table 2), which is above the minimum threshold recovery of 85% for an ideal bioink, as suggested by Kiyotake et al. [53]. Moreover, the obtained values are considerably higher than the ones reported by Olmos-Juste et al. [52] for alginate-NFC inks (between 66 and 72%), for which the rheological behavior is influenced by high NFC contents and not by a pre-crosslinking step [52]. These results confirmed that all alginate-LNF inks showed proper recovery properties to be processed by extrusion printing and for production of printed constructs with high resolution and shape-fidelity, without the use of a CaCl_2_ printing bath, a strategy commonly employed to promote instant gelation and to increase the shape fidelity in the printing process of alginate-based bioinks [29].

### 3.2. Characterization of the Alginate-LNF Hydrogels

Afterwards, fully crosslinked alginate-LNF hydrogels were prepared, using the ink formulations previously developed, and characterized in terms of their morphology, swelling behavior, degradation profiles in different media, mechanical performance (including rheological studies) and cytotoxicity towards human keratinocyte (HaCaT) cells, to have a first insight into the performance and properties of 3D-printed constructs once they are fully crosslinked after the printing process.

#### 3.2.1. Morphology of the Fully Crosslinked Alginate-LNF Hydrogels

**Figure 3:**
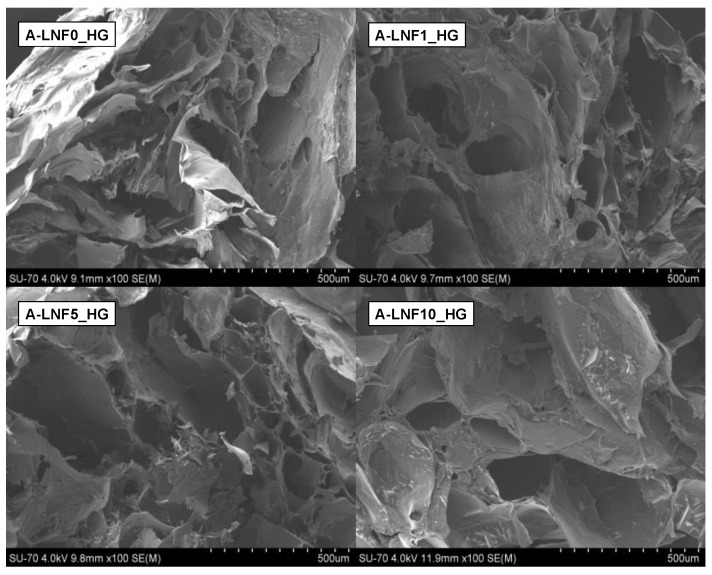
SEM micrographs of the surface of the freeze-dried fully crosslinked alginate-LNF hydrogels with different contents of LNFs.

The morphological characterization of the freeze-dried alginate-LNF hydrogels was investigated by SEM, and the retrieved micrographs are shown in Figure 3. These images revealed that all samples present an interconnected porous microstructure, in accordance with previous reports on the morphology of alginate-based hydrogels [56]. This type of morphologic arrangement is essential to produce cell-laden scaffolds, as it allows nutrient transport within the scaffold and between the scaffolds and their surroundings [7].

Moreover, it can also be observed that the increase in the content of LNFs led to pores with larger dimensions (with A-LNF0_HG showing 31.479 ± 13.431 μm; A-LNF1_HG: 54.701 ± 7.547 μm; A-LNF5_HG: 94.082 ± 18.011 μm; A-LNF10_HG: 136.093 ± 31.581 μm) and, therefore, to a less compact structure. The porosity of hydrogels depends on the ratio of solid content and their amount of water [57]. As the variance of the solid content between all the formulations is not very significant, it can be concluded that the porosity of these alginate-LNF hydrogels is more dependent on their water content, which is probably related with their higher ability to absorb water with the increasing amount of LNFs, as will be discussed below. This less compact porous structure of the hydrogels with LNFs is highly beneficial for cell growth and proliferation [55,58,59].

#### 3.2.2. Swelling Behavior

**Figure 4:**
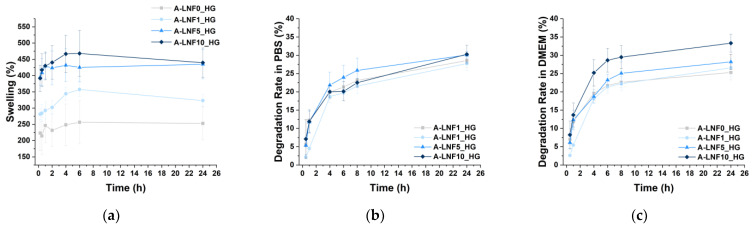
(**a**) Swelling profile in ultrapure water at 37 °C, (**b**) degradation rates in PBS at 37 °C, and (**c**) degradation rates in DMEM at 37 °C for alginate-LNF hydrogels with different contents of LNFs. All assays were performed with *n* = 3.

As previously referred, hydrogels are materials with the ability to absorb and retain high amounts of water [60,61]. This feature of hydrogels is quite advantageous for application in the bioprinting of living tissues, as it is related with the native tissue microenvironment mimicry [62]. The swelling ratio of freeze-dried alginate-LNF hydrogels, placed in ultrapure water at 37 °C, was assessed during 24 h based on the amount of absorbed water (Figure 4a), and the results obtained showed that all samples present high swelling ratios (viz. A-LNF0_HG: 256.48 ± 65.97%; A-LNF1_HG: 357.35 ± 22.12%; A-LNF5_HG: 425.19 ± 43.87%; A-LNF10_HG: 468.16 ± 70.71%), reaching a swelling equilibrium after about 6 h. It is well-known that the swelling ratio of hydrogels significantly depends on the type of polymer, its content, and the crosslinking method, parameters that are also related with the crosslinking degree and gel porosity [7,61,63]. As the concentrations of alginate and of the crosslinking CaCl_2_ solution used were the same for all samples, this test intended to evaluate the influence of the LNF content on the swelling ratio of the alginate hydrogels. Indeed, results showed that the swelling ratio of the alginate-LNF hydrogels increased for higher LNF content. After 24 h, the swelling ratios of A-LNF5_HG (434.91 ± 45.22%) and A-LNF10_HG (439.66 ± 40.23%) were significantly higher than those of A-LNF0_HG (252.95 ± 50.66%) and A-LNF1_HG (323.18 ± 18.41%). Increased swelling ratios can be attributed to lower crosslinking densities for the hydrogels with higher concentration of LNFs. These results are also in line with the SEM observations that revealed less compact porous structures with the increasing of the LNF content. These outcomes differ from those of the work of Olmos-Juste et al. [52], where lower swelling degrees were reported for increasing concentrations of cellulose nanofibers (NFC) in alginate-NFC hydrogels. However, in this case, apart from the fact that higher NFC contents were used (similar to those of alginate), the hydrogels were not crosslinked.

Overall, these data suggest that the swelling ratio of the alginate-LNF hydrogels can be tuned with the adjustment of the LNF content, and that they are suitable for 3D-bioprinting of living constructs.

#### 3.2.3. Degradation Profile of the Hydrogels

The degradation profile of the alginate-LNF hydrogels was evaluated due to the importance of matching the degradation rate of the printed living constructs with the rate at which native ECM and neo-tissues are produced [64,65,66]. The degradation profiles of all samples in PBS at 37 °C (Figure 4b), that mimic physiological conditions, and in complete DMEM medium also at 37 °C (Figure 4c), that simulate the incubation conditions after bioprinting, were retrieved by monitoring the weight changes of the hydrogels over 24 h. The degradation profiles of all hydrogels in PBS are very similar; after a rapid disintegration phase during the first 6 h of incubation, the degradation rates reached a more stable phase. After 24 h of incubation, the observed weight losses were also very similar for all investigated samples, namely A-LNF0_HG 27.72 ± 1.91%; A-LNF1_HG 28.55 ± 1.91%; A-LNF5_HG 30.07 ± 0.89% and A-LNF10_HG: 30.27 ± 2.45%. Olmos-Juste et al. [52] reported higher degradation rates in PBS for alginate-NFC hydrogels (up to 50%); however, these hydrogels were not crosslinked with CaCl_2_. The degradation of crosslinked alginate hydrogel in PBS is related to ionic exchange which causes the displacement of the Ca^2+^ cations, disrupting the hydrogel network [28].

In DMEM, higher weight losses were observed for the samples with higher LNF contents, namely A-LNF5_HG (28.19 ± 2.08%) and A-LNF10_HG (33.31 ± 2.41%). Similar degradation rates for crosslinked hydrogels composed of alginate (3 wt.%), carboxymethylcellulose (3 wt.%) and NFC (1.5 wt.%) in culture media were reported by Zidaric et al. [67], with weight loss values of 30% after 24 h of incubation. This degradation is also associated with network disruption related to the cationic exchange between Ca^2+^ and other ions present in media. The higher degradation rates for the hydrogels with higher LNF content could be associated with interactions of LNFs with media that have a more complex composition than PBS.

#### 3.2.4. Mechanical Performance

**Figure 5:**
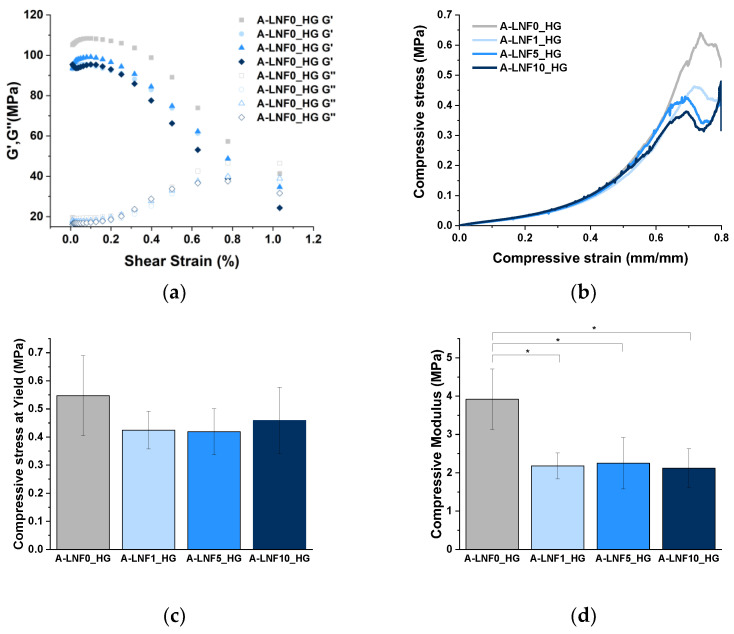
(**a**) G′ and G″ as a function of the shear strain, (**b**) the compressive stress curves (**c**) compressive stress at yield and (**d**) compressive modulus for alginate-LNFs with different LNF contents. The statistical differences were analyzed using ANOVA Tukey test (* *p* < 0.05).

The mechanical properties of 3D-printed constructs strongly influence their shape fidelity and the cellular mechanical support after the printing process [68,69]. Therefore, the mechanical properties of the fully crosslinked alginate-LNF hydrogels were assessed firstly by rheological characterization. Oscillatory shear strain measurements were carried out over the range 0 to 100% strain at a fixed frequency of 1 Hz (Figure 5a). As expected, all samples revealed higher G′ than G″, which indicates that all alginate-LNF hydrogels are solid-like biomaterials with the prevalence of rheological elastic behavior over viscous behavior, as previously reported for alginate-based hydrogels [29,68]. However, it was perceived that the increase in the LNF content led to a slight decrease in the initial G′ at 0,1% strain, certainly due to a lower crosslinking density as previously discussed for the morphology and swelling behavior.

The compressive mechanical properties were also measured for all samples (Figure 5b–d), and similar results were noted, as the compressive modulus value of the alginate hydrogel decreased with the addition of LNFs. However, no significant differences were perceived between the hydrogels with different contents of LNFs. Regarding the compressive stress at yield, no differences were observed between the alginate and alginate-LNF hydrogels.

Altogether, these results suggest that the degree of crosslinking of alginate polymeric chains is decreased with the addition of LNFs, impacting their general properties, and their tuning could be used to adjust the performance of the hydrogels.

#### 3.2.5. In Vitro Cytotoxicity towards HaCaT Cells

**Figure 6:**
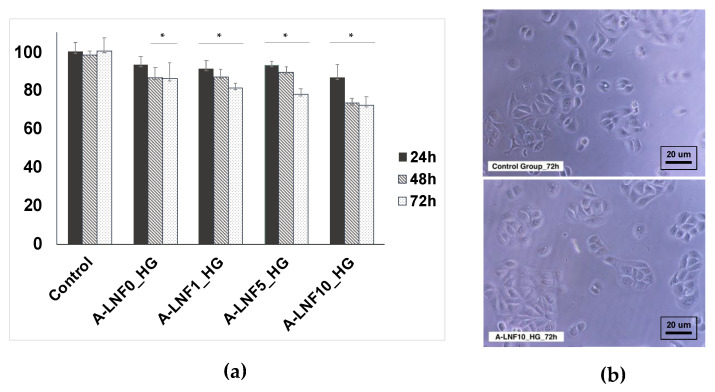
(**a**) Cell viability at 24 h, 48 h and 72 h of exposure and (**b**) optical micrographs of the HaCaT cells after 72 h of exposure to A-LNF10_HG compared to the control group. The statistical differences were analyzed using ANOVA Tukey test (the symbol * denotes the means with a significant difference from the control *p* < 0.05).

The in vitro cytotoxicity of the alginate-LNF hydrogels was evaluated in human HaCaT keratinocyte cells through the indirect MTT assay [70]. The use of HaCaT cell line has been reported in various works, singularly or in combination with fibroblast cells, for the development of bioprinted skin models [71,72]. HaCaT cells’ metabolic activity after 24, 48 and 72 h of exposure to alginate-LNF hydrogels extracts is represented in Figure 6a. Cell viability was maintained above the 70% threshold [73] for all samples after every exposure time, confirming their non-cytotoxicity towards this cell line and their suitability for being loaded with cells. The highest cell viability values, closer to the negative control, were achieved after 24 h of exposure to all hydrogel extracts (A-LNF0_HG: 93 ± 5%; A-LNF1_HG: 91 ± 4%; A-LNF5_HG: 93 ± 2%; A-LNF10_HG: 87 ± 6%). However, after 72 h the cell viabilities are still high and the cells maintained their typical morphology, as illustrated for the cells exposed to the inks with a higher content of LNFs (Figure 6b). These results were expected, as sodium alginate is highly recognized as a non-cytotoxic biopolymer for different cell lines [25,27], including HaCaT cells [74]. To the best of our knowledge, the cytotoxicity of LNFs towards HaCaT cells has not yet been evaluated; however, other works have proven their non-cytotoxicity towards other skin cell lines, such as L929 fibroblasts [44], also widely used in skin models.

### 3.3. Optimization of the Printing Parameters and Printability of Alginate-LNF Inks

**Figure 7:**
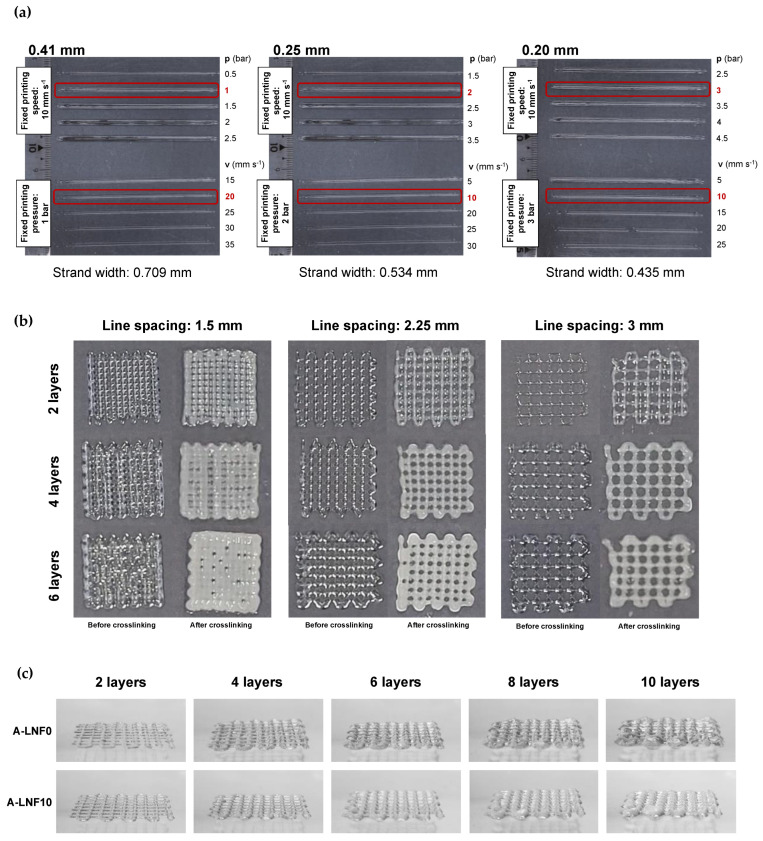
Optimization of the (**a**) printing parameters, namely, nozzle diameter (0.41, 0.25 and 0.20 mm), printing pressure and speed; (**b**) 3D scaffold model with different line spacing (1.5, 2.25 and 3 mm) and number of layers (2, 4 and 6 layers) for alginate-LNF0 ink; and (**c**) side-view of 3D scaffold models obtained using the A-LNF0 and A-LNF10 bioinks (before crosslinking), with varying number of layers (2, 4, 6, 8 and 10 layers) with a line spacing of 2.25 mm.

The influence of the extrusion 3D-printing parameters, namely nozzle diameter, printing pressure and printing speed, on the quality of single printed lines using the newly developed inks was studied. As the rheological properties of all the inks were very similar, the A-LNF0_Ink was used for these preliminary printing tests. The temperature of the printing cartridge was set at 20 °C for all experiments. First, printing pressure and speed were adjusted for nozzles with three different diameters (0.41, 0.25 and 0.20 mm) (Figure 7a). The printing pressure is one of the most important parameters, because it has an influence on the printing output and the printed strand width. To select the adequate printing pressure, the strand’s width was measured and based on the obtained results (Figure 7a), the selected printing pressures for the 0.41-, 0.25- and 0.20-mm nozzles were of 1, 2 and 3 bar, respectively. Considering the selected pressures for each nozzle diameter, the strand width of the printed hydrogels was further adjusted by varying the printing speed (Figure 7a). The obtained results allow one to set the printing speed to 20 mm s^−1^ for the 0.41-mm nozzle and at 10 mm s^−1^ for the 0.25- and 0.20-mm nozzles. As a general indication, high pressures not only result in a larger diameter of printed lines but may also compromise the cell viability due to higher shear stresses on cells during the printing process, which can result in cell death because of the disruption of the cell membrane [75]. On the other hand, high printing speeds reduce the printing time and lead to lower strand diameter but may result in the tearing of strands during extrusion [56]. Therefore, a balance between these two parameters is critical to ensure good printing resolution and high cell viability. Considering this premise, the selected optimal printing parameters for the conditions investigated were a nozzle diameter of 0.25 mm, printing pressure of 2 bar and printing speed of 10 mm s^−1^.

Afterwards, 3D grid like model scaffolds with 20 mm × 20 mm were printed also using the A-LNF0_Ink formulation and the printing conditions previously selected, and for crosshatch infill line spacing, between 1.5 and 3 mm, and different layer numbers, between 2 and 6 (Figure 7b), to define the best characteristics of the printed scaffold models for these inks. The printed scaffolds were fully crosslinked to qualitatively evaluate this final step on the shape stability of the printed structures. It was observed that only the scaffolds with line spacing of 1.5 mm showed low shape fidelity, and this was preeminent with an increase in layer number and after crosslinking. To balance the shape fidelity and resolution of the structures, the line spacing selected for further studies was 2.25 mm.

**Figure 8:**
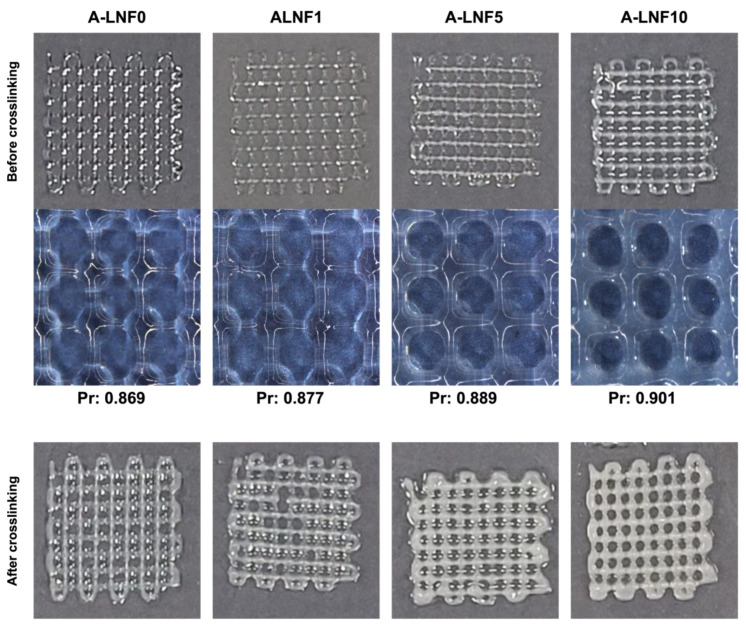
Printed scaffolds with 2 layers of the alginate-LNF inks formulations with different LNF contents.

After the optimization of the printing parameters and the 3D printed scaffold model characteristics, these were used to print 3D scaffolds with the A-LNF ink formulations. In the sideview images of constructs printed with the ink with 10% of LNFs (A-LNF10) and the ink without LNFs (A-LNF0), and with up to 10 layers (Figure 7c), it is visible that the shape fidelity of the constructs increases with the content of LNFs in the inks. In fact, for the constructs with 8 and 10 layers, the shape fidelity of the construct printed with the A-LNF0 is visibly lower than that with LNFs (A-LNF10). This is also perceptible for all 3D printed constructs obtained with the inks with different LNF contents and with 2 printed layers (Figure 8). 

To corroborate the visual analysis of the 3D-printed structures, the printability of the inks was determined, as it is strongly related to the shape fidelity of the printed structures. When an extruded filament possesses an ideal printability (*Pr* = 1), the filament will present a constant 3D width which results in the construction of regular grids with defined square pores. In the case of under gelation (*Pr* < 1), a slurry filament is printed, and pores develop a more circular form due to layer fusing. In the case of over-gelation (*Pr* > 1), scaffold layers do not fuse, and the stability of the structure is compromised [76]. The printability (*Pr*) was calculated by measuring the grid pore diameter and the grid pore area of the grid scaffolds after printing and before the final crosslinking. For all the ink formulations, the *Pr* was about 0.9, a value within the reported acceptable range of 0.9–1.1 [76]. The printability of the A-LNF0 is in accordance with that reported by Datta et al. [77] for a 5% alginate (*w*/*v*) bioink (0.88). However, a slight increase in the printability of the alginate hydrogels was observed with the amount of LNFs. This enhancement is certainly related to the nanofibrillar morphology of the LNFs that helps mechanically support the printed structure. Improvements in the printability of alginate hydrogels by the incorporation of nanocellulose fibrils have also been reported [78].

On the other hand, one of the advantages of the use of a pre-crosslinking step to modulate the rheology of the alginate-LNF inks is the avoidance of the use of a CaCl_2_ printing bath to ensure the constructs’ shape fidelity. The retrieved printability of the alginate-LNF ink formulations also confirms this fact, since all the constructs maintained their shape fidelity after printing, with no significant differences observed after the final crosslinking (Figure 8).

### 3.4. Bioprinting of Cell-Laden Alginate-LNF Bioinks

**Figure 9:**
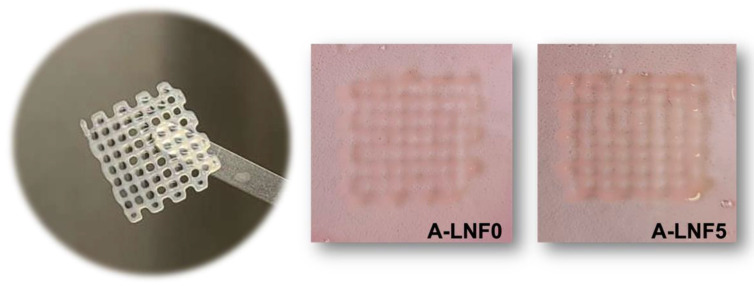
Bioprinted cell-laden scaffolds of A-LNF0 and A-LNF5 bioinks in culture media.

Finally, HaCaT cells were loaded in the alginate-LNF hydrogel formulations aiming at bioprinting 3D living constructs and studying the effect of the bioprinting process on cell viability using these bioinks. The A-LNF5 formulation was selected for this assay because of its intermediate composition regarding the LNF content. The A-LNF0 formulation was also used for comparison purposes, to evaluate the influence of LNFs on the cell viability. These bioinks were printed using the conditions and scaffold model characteristics previously defined, and no significant differences on the shape fidelity and resolution were observed when compared with the constructs without cells, as shown in Figure 9.

**Figure 10:**
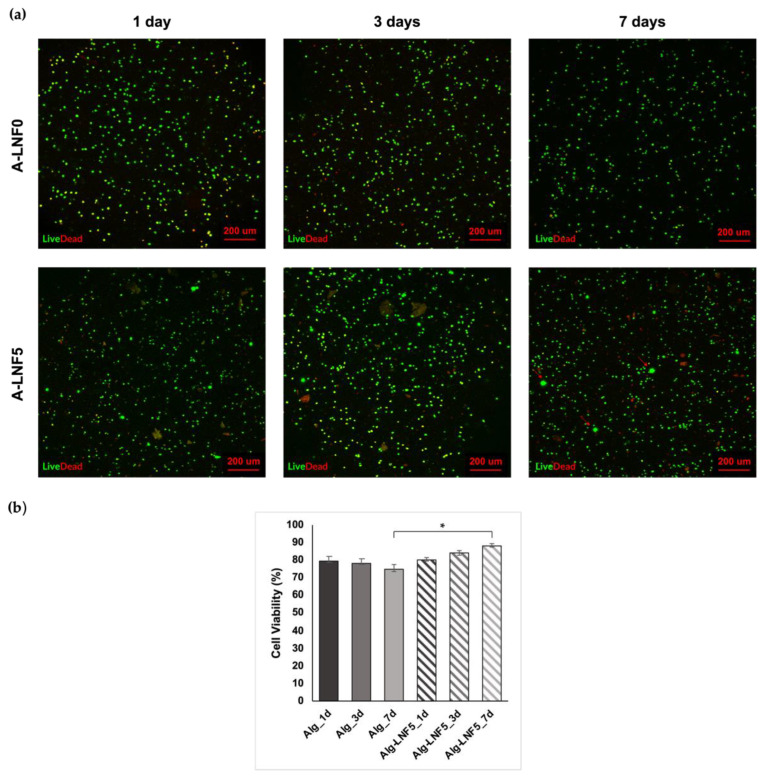
HaCaT cell viability on alginate-LNF bioprinted scaffolds (**a**) representative LIVE/DEAD fluorescence micrographs of HaCaT cells encapsulated within the A-LNF0 and A-LNF5 bioinks after 1, 3 and 7 days. (**b**) cell viability percentage at 1, 3 and 7 days after bioprinting. The statistical differences were analyzed using ANOVA Tukey test (* *p* < 0.05).

The evaluation of the cell viability within the A-LNF0 and A-LNF5 bioprinted scaffolds was carried out by performing live/dead staining at day 1, 3 and 7. As it can be observed in the fluorescence micrographs of the printed scaffolds (Figure 10a), cells were homogeneously distributed within the scaffolds with cell survival of up to 7 days, for both formulations. HaCaT cell viability at day 1, day 3 and day 7 (Figure 10b) was calculated based on the ratio between live (in green) and dead (in red) cells. Cell viabilities on day 1 after bioprinting were similar for both scaffolds, namely 79.32 ± 2.54% for A-LNF0 and 80.09 ± 1.38% for A-LNF5. Habib et al. [56] reported lower cell viabilities (around 60%) at 24 h post-bioprinting for pancreatic cancer cells loaded in 4% (*w*/*v*) alginate and 4% (*w*/*v*) alginate-4% (*w*/*v*) CMC bioinks. This difference could be related to the different cell line used and the printing conditions. Therefore, our results indicate that the selected printing conditions, namely the nozzle diameter, the printing pressure and the bioink composition (alginate and LNFs), were suitable to maintain a high cell viability after the bioprinting process. At 7 days post-bioprinting, it was observed that the cell viability on the A-LNF0 scaffolds slightly decreased (74.60 ± 2.40%), whereas it increased in the A-LNF5 scaffolds (87.99 ± 1.28%). Moreover, in the micrographs of the bioprinted scaffolds with LNFs (Figure 10a), the formation of cell aggregates is also perceived, which might be further indicative of an increase in cell proliferation for the scaffolds with LNFs [79]. The increased cell viability and the formation of the cell aggregates suggests that the addition of LNFs to the alginate bioinks promotes an enhancement of their biological properties and overcomes one of the main limitations of alginate bioinks, viz. their considerable biological inertness. Similar results were obtained by Kim et al. [50], with the addition of 3 wt.% of silk fibroin to a 3 wt.% alginate-based bioink loaded with NIH-3T3 fibroblast cells, showing improved cell viability at 7 days after bioprinting (up to 98%).

In sum, these results confirm that the selected bioprinting conditions and the alginate hydrogel bioinks with LNFs are adequate to produce living constructs with high cell viability for exploitation in different fields.

## 4. Conclusions

This study aimed to develop novel alginate-LNF nanocomposite hydrogel-based bioinks for 3D bioprinting applications. An alginate solution 4% (*w*/*v*) and alginate-LNF suspensions with varying LNF content (1, 5 and 10 wt.%) were pre-crosslinked with CaCl_2_ 0.5% (*w*/*v*) and their rheological behavior was investigated. Despite the slight improvement in rheological parameters perceived with the addition of LNFs, the pre-crosslinking of the alginate-LNF suspensions has a main role in the optimization of their shear-thinning behavior and to produce suitable ink matrices for extrusion printing. Moreover, all ink formulations presented excellent recovery ability (~90%), indicating suitable properties to ensure post-printing shape fidelity without the use of a Ca^2+^ enriched printing bath.

Fully crosslinked hydrogels of all formulations that mimic 3D-printed scaffolds were prepared with CaCl_2_ 2% (*w*/*v*), and characterized in terms of their morphology, swelling capacity and degradation in different media, and mechanical performance. The prepared hydrogels showed an increased water-swelling capacity and disintegration rate in DMEM, and decreased compressive modulus, with the rise in the amount of LNF. These results are related to the morphological features of these hydrogels, which present a less compact structure with increases in the content of LNFs. This morphology can be considered very suitable for the accommodation and growth of living cells. Furthermore, these hydrogels were found to be non-cytotoxic towards human keratinocytes (HaCaT cell line).

Following this, the printing conditions (printing parameters and 3D scaffold model) were optimized using the A-LNF0 formulation as a model ink. The printability of all alginate-LNF inks was evaluated after printing a 20 mm × 20 mm grid-like scaffold with a line spacing of 2.25 mm with the optimal printing parameters (nozzle diameter: 0.25 mm; printing pressure: 2 bar; and printing speed: 10 mm s^−1^). The alginate-LNF inks showed good printability (*Pr*: ~0.9) with a slight increase perceived with the increasing amount of LNFs.

As a proof of concept, the formulation A-LNF5 (and the A-LNF0 for comparison purposes) were loaded with HaCaT cell (with final cell density of 2 × 10^6^ cells mL^−1^) to print cell-laden scaffolds. Their cell viability was evaluated for 1, 3 and 7 days after bioprinting. The LIVE/DEAD fluorescence micrographs showed that the cells were homogeneously distributed within the scaffolds with similar cell viabilities on day 1 after bioprinting (~80%). After 7 days the cell viability on the A-LNF0 scaffolds slightly decreased (74.60 ± 2.40%), whereas it increased in the A-LNF5 scaffolds (87.99 ± 1.28%). Therefore, the addition of LNFs to the alginate bioinks promotes an enhancement of their biological properties.

Overall, these results confirm that the developed alginate-LNF hydrogel bioinks showed improved rheological behavior and printability, and that the selected bioprinting conditions are adequate to produce living constructs with high cell viability for exploitation in different bioprinting applications.

## Figures and Tables

**Table 1:** Identification and composition of the alginate-LNF suspensions, inks, and hydrogels.

Sample	Alginate% (*w*/*v*)	LNFs wt.%	CaCl_2_% (*w*/*v*) Pre-Crosslinker	CaCl_2_% (*w*/*v*) Full-Crosslinker
Alginate-LNF suspensions				
A-LNF0	4	--	--	--
A-LNF1	4	1	--	--
A-LNF5	4	5	--	--
A-LNF10	4	10	--	--
Alginate-LNF Inks				
A-LNF0_Ink	4	-	0.5	--
A-LNF1_Ink	4	1	0.5	--
A-LNF5_Ink	4	5	0.5	--
A-LNF10_Ink	4	10	0.5	--
Alginate-LNF Hydrogels				
A-LNF0_HG	4	--	0.5	2
A-LNF1_HG	4	1	0.5	2
A-LNF5_HG	4	5	0.5	2
A-LNF10_HG	4	10	0.5	2

**Table 2:** Viscosity at 0.1 s^−1^ shear rate, power-law fitting parameters, yield stress and recovery percentage of the alginate-LNF suspensions and alginate-LNF inks with different LNF contents.

Sample	Viscosity (Pa·s)	*n*	K	R^2^	Yield Stress (Pa)	Recovery (%)
Alginate-LNF suspensions						
A-LNF0	0.722 ± 0.203	0.939 ± 0.037	0.636 ± 0.005	0.948	--	--
A-LNF1	0.861 ± 0.200	0.940 ± 0.005	0.720 ± 0.008	0.916	--	--
A-LNF5	1.056 ± 0.369	0.927 ± 0.004	0.937 ± 0.009	0.949	--	--
A-LNF10	1.250 ± 0.338	0.921 ± 0.005	1.019 ± 0.010	0.945	--	--
Alginate-LNF Inks						
A-LNF0_Ink	271.50 ± 29.85	0.322 ± 0.004	56.924 ± 0.532	0.999	50.32 ± 0.49	92.599 ± 4.782
A-LNF1_Ink	274.23 ± 49.36	0.397 ± 0.002	69.269 ± 1.058	0.998	55.14 ± 0.83	89.783 ± 5.028
A-LNF5_Ink	281.67 ± 85.71	0.339 ± 0.005	60.807 ± 0.557	0.997	58.01 ± 1.68	89.328 ± 2.402
A-LNF10_Ink	336.10 ± 84.55	0.316 ± 0.003	69.196 ± 0.425	0.999	65.63 ± 2.84	91.460 ± 4.719

## Data Availability

The data presented in this study are available in this article.
